# Characterization of Ugandan Endemic *Aspergillus* Species and Identification of Non-Aflatoxigenic Isolates for Potential Biocontrol of Aflatoxins

**DOI:** 10.3390/toxins14050304

**Published:** 2022-04-26

**Authors:** Godfrey Wokorach, Sofie Landschoot, Amerida Lakot, Sidney Arihona Karyeija, Kris Audenaert, Richard Echodu, Geert Haesaert

**Affiliations:** 1 Department of Plants and Crops, Campus Schoonmeersen Building C, Faculty of Bioscience Engineering, Ghent University, Valentin Vaerwyckweg 1, B-9000 Ghent, Belgium; godfrey.wokorach@ugent.be (G.W.); sofie.landschoot@ugent.be (S.L.); kris.audenaert@ugent.be (K.A.); 2 Multifunctional Research Laboratories, Gulu University, Gulu P.O. Box 166, Uganda; lakotamerida@gmail.com (A.L.); sidneykary@gmail.com (S.A.K.); richardechodu2009@gmail.com (R.E.); 3 Department of Biology, Faculty of Science, Gulu University, Gulu P.O. Box 166, Uganda

**Keywords:** *Aspergillus flavus*, *Aspergillus niger*, *Aspergillus tamarii*, aflatoxin B1

## Abstract

Acute stunting in children, liver cancer, and death often occur due to human exposure to aflatoxins in food. The severity of aflatoxin contamination depends on the type of *Aspergillus* fungus infecting the crops. In this study, *Aspergillus* species were isolated from households’ staple foods and were characterized for different aflatoxin chemotypes. The non-aflatoxigenic chemotypes were evaluated for their ability to reduce aflatoxin levels produced by aflatoxigenic *A. flavus* strains on maize grains. *Aspergillus flavus* (63%), *A. tamarii* (14%), and *A. niger* (23%) were the main species present. The *A. flavus* species included isolates that predominantly produced aflatoxins B1 and B2, with most isolates producing a high amount (>20 ug/µL) of aflatoxin B1 (AFB1), and a marginal proportion of them also producing G aflatoxins with a higher level of aflatoxin G1 (AFG1) than AFB1. Some non-aflatoxigenic *A. tamarii* demonstrated a strong ability to reduce the level of AFB1 by more than 95% when co-inoculated with aflatoxigenic *A. flavus.* Therefore, field evaluation of both non-aflatoxigenic *A. flavus* and *A. tamarii* would be an important step toward developing biocontrol agents for mitigating field contamination of crops with aflatoxins in Uganda.

## 1. Introduction

Some fungi belonging to the genus *Aspergillus*, e.g., *A. flavus, A. parasiticus*, and *A. nomius* produce aflatoxins [1]. Aflatoxins are a food safety hazard that is classified among class I human carcinogens [2]. Prolonged exposure to aflatoxins at a dose above acceptable limits (4–30 ng/µL) usually results in acute hepatic necrosis and death [3,4,5]. Cases of aflatoxin fatality in Uganda have been reported due to ingestion of food that has been highly contaminated with aflatoxin [6]. The high incidence of hepatitis B, together with frequent exposure to aflatoxins, has elevated the risk of hepatoma in Uganda. Additionally, high exposure to aflatoxins during pregnancy often results in infants born with reduced birth weight [7]. Infant exposure to high aflatoxin either in breast milk or supplementary feeds is manifested with growth impairment, reduced immunity, and protein malnutrition [8]. As such, aflatoxin exposure is regarded as a contributing factor to a high stunting rate between 35–40% among infants in Uganda. Generally, more than 90% of the individuals in Uganda get exposed to high aflatoxin levels, which are detectable in their body fluids [9,10]. High exposure is due to relying on crops like maize, sorghum, and groundnuts that are highly susceptible to aflatoxin contamination. In some areas, nearly 100% of cereal grains and groundnuts get contaminated with aflatoxin [11]. Most household grains get contaminated with aflatoxin concentrations exceeding regulatory limits of 10 µg/Kg. Besides the health issues, the economic losses due to non-tariff trade barriers from aflatoxin contamination of agricultural products is estimated to be around US$577 million per year [12]. The etiology for heavy contamination of food and feeds in Uganda with aflatoxins is due to the high temperature and humidity and poor agricultural practices, which promote growth and proliferation of aflatoxigenic *A. flavus* and *A. parasiticus* [13].

Several classes of aflatoxins have been documented, and the most commonly detected are aflatoxin B1, B2, G1, and G2 [14]. The classes of aflatoxins detected in food commodities depend on the resident/native aflatoxin producing fungal genotypes [15]. Species within *Aspergillus* section *Flavi* are morphologically characterized as one of two morphotypes, S-type or L-type, based on the size of their sclerotia [16], and there are expected levels of aflatoxin production associated with each morphotype [17]. On average, isolates that belong to the S-type group are characterized by the production of a large quantity of B-aflatoxins, whereas the L-type produces variable quantities of B-aflatoxins [18]. An S-type taxon referred to as SBG mostly occurs in West Africa and produces large amounts of both aflatoxins B and G, whereas SB variants produce only B aflatoxins and are found to be dominant in the USA [19]. Other variants geographically restricted to western Kenya produce high amounts of lethal aflatoxins responsible for periodic outbreaks of acute aflatoxicosis in that region [20]. However, there are *Aspergillus* species (including strains of *A. flavus*) that naturally do not produce aflatoxins [21].

Contamination of food items with aflatoxins begins in the field before crops are harvested and continues through storage [22]. Once pre-harvest contamination has occurred, post-harvest handling, such as proper drying of grains, can only limit further contamination during storage. Complete removal of aflatoxins from food is very challenging once pre-harvest contamination has occurred in the field. However, some physical and chemical treatments and food processing techniques can lower the aflatoxin concentrations to a relatively safe level [23]. Physical treatment of grains with pulsed light reduces aflatoxins in food samples by over 90% [24]. Additionally, chemical treatments with ozone and ammonia are also among the approaches for decontamination of aflatoxins, especially in animal feeds [25]. The methods used for decontamination of aflatoxins in food are impractical to adopt in low to middle income countries due to limited financial resources.

In Uganda and most of Africa, much focus has been on traditional methods of pre-harvest and post-harvest aflatoxin management, such as the use of safe storage bags, proper drying of grains, crop rotation, sorting, and milling [26,27]. Such pre-harvest and post-harvest management techniques appear to be ineffective at properly managing aflatoxin contamination of grain, as recurrent high contamination levels of grains with aflatoxin are constantly being reported. Many countries have achieved aflatoxin reduction between 60 and 100% by applying non-aflatoxigenic variants of *A. flavus*. Evidence suggests that these biocontrol agents can displace the aflatoxigenic variants in crop fields by competitive exclusion, thereby reducing the population of aflatoxigenic genotypes in the field and reducing the risk of high levels of pre-harvest aflatoxin contamination [28]. The applicability of this method was first demonstrated in the USA to control aflatoxins in cotton fields [17]. Currently, many countries in sub-Saharan Africa, such as Kenya, Tanzania, Gambia, Nigeria, and Senegal, have demonstrated the applicability of non-aflatoxigenic (AF−) variants in reducing crop contamination with aflatoxins [29]. The benefit of non-aflatoxigenic strains is threefold: (i) it reduces aflatoxins in the field; (ii) the protection can continue during storage; and (iii) the protection can last for more than one season in the field and thus reduces the costs for continued intervention. However, it greatly relies on the discovery, testing, and use of native non-aflatoxigenic strains. Consequently, each country has to develop its biological control from the pools of native non-aflatoxigenic variants that have evolved and adapted to thrive in the local environment. Therefore, the objectives of this study were: (i) to characterize native *Aspergillus* population within northern Uganda and profile their aflatoxin production capacities, and (ii) to evaluate the non-aflatoxigenic variants for their ability to suppress aflatoxin production among aflatoxigenic (AF+) isolates on maize grains in a laboratory setting to guide the selection of candidate biocontrol isolates for further field evaluation.

## 2. Results

### 2.1. Characterization of Aspergillus Species

**Figure 1:**
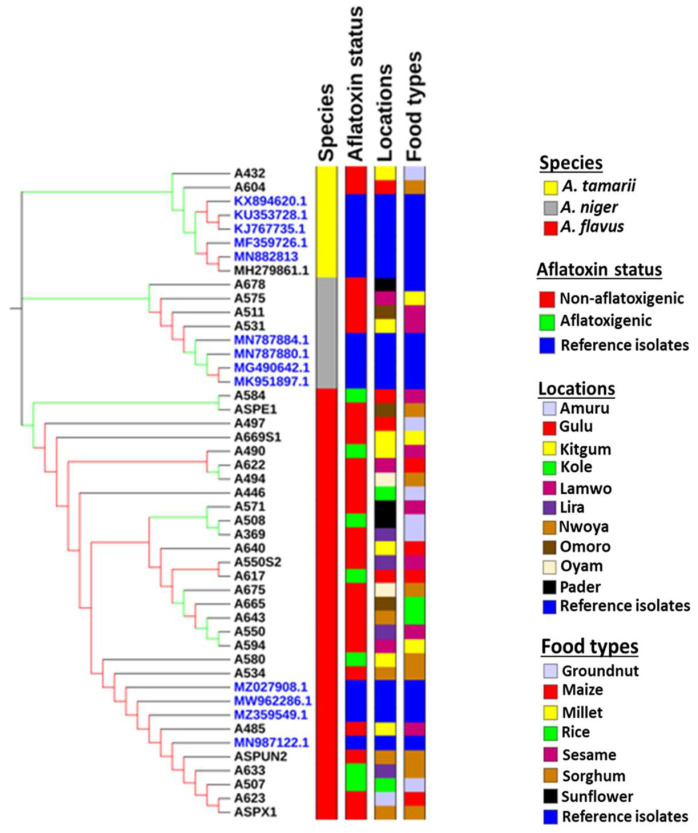
Phylogenetic tree showing cluster separation between *A. flavus*, *A. tamarii*, and *A. niger*. Green branch length shows nodes with a 100% bootstrap value and those with red branch length show a bootstrap value of less than 100%. The leaves with blue color are reference isolates and those with black color are *Aspergillus* isolates from this study. Aflatoxin status, sample locations, and food types of reference isolates were not determined and not presented in the figure.

Molecular characterization using partial sequences of the calmodulin gene classified the isolates into three species: *Aspergillus niger*, *Aspergillus flavus*, and *Aspergillus tamarii* (Figure 1). Both *A. flavus* and *A. tamarii* belong to the *Aspergillus* section *Flavi* group. Morphological characteristics show that all the isolates of *A. tamarii* were brown, *A. flavus* isolates were green and *A. niger* isolates appeared black when grown on both PDA and YES media. Among the *Aspergillus* fungi that were isolated, the proportion of *A. flavus* isolates (62.96%) was significantly higher than the proportion of *A. niger* (23.46%) isolates and *A. tamari* (13.58%) isolates. A phylogenetic tree was inferred from a partial sequence of calmodulin gene and showed *A. tamarii* and *A. flavus* diverge to form two different sub-clades, whereas *A. niger* forms a distantly related phylogenetic clade (Figure 1). No clustering of *A. flavus* based on the AF+ or AF− chemotype was observed. This was the same for sample types and locations from which the samples were collected (Figure 1).

### 2.2. Distribution of Aspergillus Species by Food Types

Among the cereal foods, *Aspergillus* fungi were most frequently isolated from sorghum grains (*n* = 22) and least frequently isolated from millet (*n* = 5) grains. Among the oil crops, sesame (*n* = 16) had the highest proportion of samples from which the *Aspergilli* were isolated compared with the groundnut (*n* = 10). *A. flavus* was found in varying amounts in six out of eight foods examined. The greatest incidence of *A. flavus* was found in the sorghum samples (18.52%), while it was not found in the cassava and sunflower samples (Table 1). The incidences of *A. niger* in our food samples were low (≤4.94% in sesame and sorghum), but this fungus was present in all eight food types (Table 1). The *A. tamarii* species was found in five food types, and its greatest incidence was in maize samples (4.94%).

### 2.3. Chemotyping of Aspergillus Isolates

**Figure 2:**
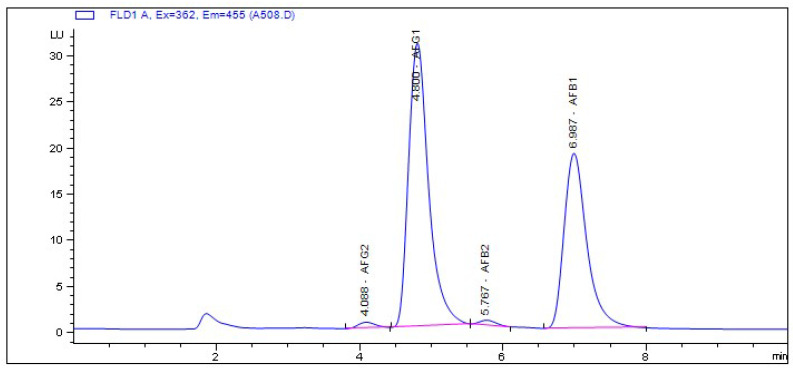
Chromatogram showing the peaks and retention time (RT) for the four aflatoxin chemotypes produced by isolate A508. The first peak is AFG2 (aflatoxin G2) with RT = 4.088 min, the second peak is AFG1 (aflatoxin G1) with RT = 4.800 min, the third peak is AFB2 (Aflatoxin B2) with RT = 5.767 min, and the fourth peak is AFB1 (aflatoxin B1) with RT = 6.967 min.

As expected, the *A. tamarii* and *A. niger* isolates did not produce aflatoxins. Additionally, a greater proportion of the isolates of *A. flavus* could not produce aflatoxins (62.75%). Only 37.25% of the *A. flavus* isolates were capable of producing aflatoxins. Evaluation of their aflatoxin chemotypes indicated no significant variation in the classes of aflatoxins produced. In fact, all but one aflatoxigenic isolate produced AFB1 and AFB2 (Table 2 and Figure S1). Although a rare occurrence for *A. flavus*, isolate A508 produced all four of the major aflatoxin classes examined (AFG2, AFG1, AFB2, and AFB1) (Table 2 and Figure 2).

**Figure 3:**
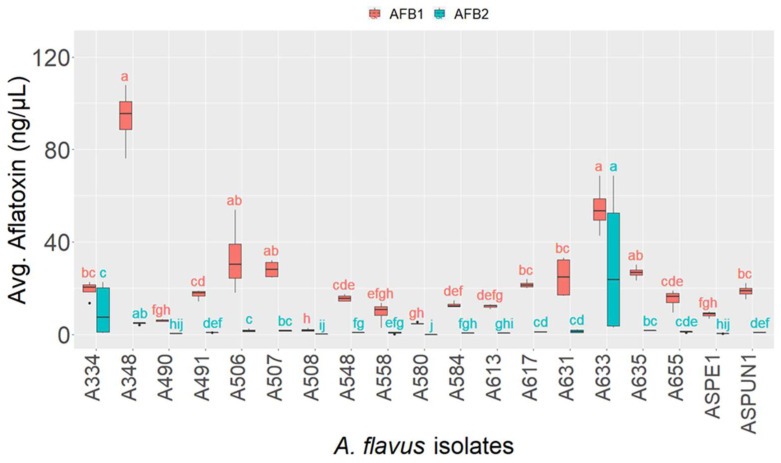
Box plot showing variation in production of AFB1 (red boxes) and AFB2 (blue boxes) for *A. flavus* isolates from multiple hosts. Different letters above the bars indicate significant differences between isolates according to a Dunn test.

There was variation in the toxicity of the different isolates with regards to the levels of AFB1 and AFB2. Generally, aflatoxigenic isolates produced a higher level of AFB1 compared to the level of AFB2 (Figure 3). Isolates A348, A633 and A506 were the most aflatoxigenic among the pool of the *A. flavus* isolated as they produced significantly higher AFB1 concentrations compared to the other isolates. Although A348 had the highest AFB1 concentration, the level did not significantly differ from that produced by isolates A633 and A506 (Figure 3). Despite producing four classes of aflatoxins, A508 was the least aflatoxigenic isolate (with regard to B-aflatoxin levels), and its concentration of AFB1 did not significantly differ from four other isolates having similarity in their toxicity potential (Figure 3). Among isolates producing moderate AFB1 levels (5–30 ng/µL), the differences were not significant.

The level of AFB2 produced by A633, A348, and A334 was significantly higher than those produced by most aflatoxigenic isolates. Although the Dunn’s test indicated their AFB2 levels to be significantly different from each other, these were not deemed significantly different in comparison to their respective AFB1 levels (Figure 3). The AFB2 levels for all other examined isolates were comparable. Only one isolate, A508, produced G aflatoxins (Figure 2). A comparison of the level of AFG1 and AFB1 produced by this isolate showed it produces nearly double the amount of AFG1 compared with AFB1. The mean level of AFG1 produced by isolate A508 was 2.53 ng/µL, whereas the mean level of AFB1 was 1.42 ng/µL indicating that it was significantly higher than (χ^2^ = 4.08, *p* = 0.04) the concentration of AFB1.

### 2.4. Testing the Biocontrol Potential of Non-Aflatoxigenic Aspergilli on Maize Grains

**Figure 4:**
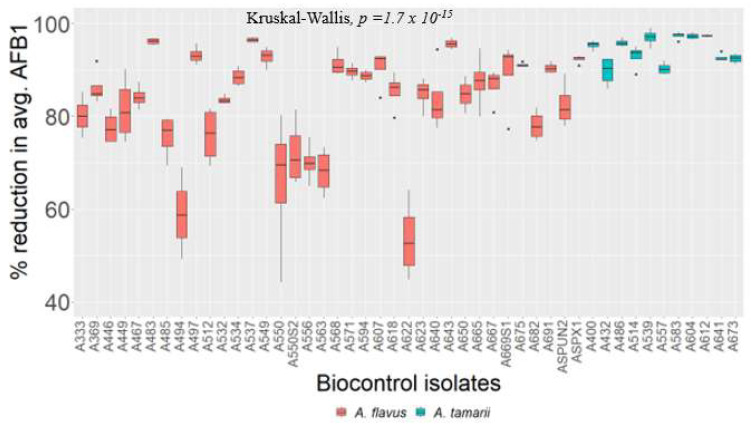
Box plot showing percent reductions on average AFB1 production by A348 after co-inoculation with non-aflatoxigenic *A. flavus* and *A. tamarii* isolates.

Maize grains (50 g) that were co-inoculated with mixtures of isolate A348 and each non-aflatoxigenic strain resulted in significantly lower levels of AFB1 compared to A348 control levels (Figure 4 and Figure S2). Generally, *A. tamarii* isolates offered more substantial reductions (>90%) of AFB1 compared to non-aflatoxigenic *A. flavus* isolates when co-inoculated with aflatoxigenic isolate A348 (Figure 4 and Figure S2). However, some *A. flavus* isolates like A483, A537 and A643 demonstrated an equally strong ability to reduce the level of AFB1 in A348. Non-aflatoxigenic isolates A622 and A494 resulted in relatively higher levels of AFB1 when co-inoculated with A348 compared with other non-aflatoxigenic isolates. The majority of non-aflatoxigenic isolates were capable of reducing the level of AFB1 ranging from 70% to 97% when co-inoculated with aflatoxigenic isolate A348 (Figure 4). Most of the isolates of *A. tamarii* resulted in a reduction in the concentration of AFB1 produced by aflatoxigenic isolate A348 by over 95%. Similarly, some isolates of *A. flavus* showed a very high reduction in the level of AFB1 concentration by over 95% (Figure 4). The least reduction in the level of AFB1 was observed with three isolates of *A. flavus* with a percent reduction between 50 and 65%.

## 3. Discussion

This study screened the natural population of *Aspergillus* in stored household food commodities for aflatoxigenic fungi to evaluate the risk of storage grain contamination with aflatoxin under sub-optimal storage conditions among farmers in Uganda. Based on colony color and molecular characterization using a portion of the calmodulin gene, the *Aspergillus* species present in sampled foods were *A. flavus*, *A. tamarii* and *A. niger*. In terms of incidence, isolates belonging to *Aspergillus* section *Flavi* were more predominant in the stored grains than *A. niger*. About 37.25% of the *A. flavus* isolates recovered from the household grains were capable of producing aflatoxins. We determined that *A. flavus* was the dominant species producing aflatoxins, whereas species like *A. parasiticus* and *A. nomius*, which have been implicated in aflatoxin contamination of grains, were not recovered from stored grains in this region. Unexpectedly, there were disproportionately more non-aflatoxigenic isolates/species recovered from the grains than aflatoxigenic isolates. Other studies indicate that incidences of aflatoxigenic and non-aflatoxigenic isolates of *A. flavus* vary between geographical locations, even within a country, with some areas favored with high incidence of aflatoxigenic strains and others having a predominance of non-aflatoxigenic strains [30]. The phylogenetic grouping was only able to infer differences between species of the fungi. No clustering of isolates was observed based on food type or sampling location. Among the *A. flavus* isolates, there was no assessment of sclerotial morphotype for our *A. flavus* strains, so no associations could be made relating to this phenotype. There also was no observable clustering based on chemotype (i.e., AF+ or AF−). In fact, isolates A508 (B + G) and A369 (AF−) were inferred to share a most recent common ancestor. Moore et al. (2017) reported observing evidence of trans-speciation and highly aflatoxigenic isolates associating with a lineage that is predominantly non-aflatoxigenic. Whether or not A369 belongs to this non-aflatoxigenic lineage remains unknown [31]. Further investigation using multiple-locus sequence typing based on beta-tubulin gene, translation elongation factor 1-alpha gene, and calmodulin gene would probably reveal insight into evolutionary differences between the AF− and AF+ *A. flavus* strains in Uganda [32,33].

Findings from aflatoxin chemotyping suggest that *A. flavus* in Uganda are predominantly non-aflatoxigenic, and that strains capable of producing aflatoxins appear mostly of the B-aflatoxin chemotype. There is even a subset of the local *A. flavus* population that produces B and G aflatoxins. However, further sampling and characterization of foods throughout Uganda are necessary to confirm this. Generally, *A. flavus* with the capacity of producing both AFBs and AFGs aflatoxins are predominantly known to occur within West Africa relative to other geographical regions whereas the genotype that predominantly produces large quantities of AFB1 and AFB2 and are known to have wider geographical dispersal including USA and Kenya [34]. Substrate type, water activity level, temperature and CO2 concentration can affect aflatoxin production [35,36]. Assessment of aflatoxin production among our *A. flavus* isolates revealed variable production of AFB1 on maize grains substrate at 30 °C. There were isolates with the ability to produce a vast amount of AFB1 on maize grains as observed in isolates A348 and A633 although most isolates produced moderate to low levels of AFB1. The presence of isolates with the ability to produce a large amount of AFB1 in household maize grains has severe implications in terms of food safety given that the mitigation strategies are not very rigorous among resource constraint subsistence farmers that form the bulk of food production in Uganda. Acute aflatoxicosis has been reported in Kenya due to the occurrence of *A. flavus* genotypes with the ability to produce very high AFB1 levels [34]. Accidental ingestion of food contaminated with isolates such as A348 and A633 with the ability to produce an exorbitant amount of AFB1 could cause acute aflatoxicosis in this region. Since aflatoxigenic *A. flavus* was isolated from framers’ stored foods, contamination of foods with aflatoxins during storage would be a common phenomenon, given the sub-optimal storage conditions common among farmers. Given that AFB1 is a highly potent carcinogenic agent compared to other aflatoxin classes, there is a need for households in Uganda to take post-harvest cautions to limit the proliferation of aflatoxigenic fungi within the storage structure. Optimizing storage conditions such as the use of hermetic storage bags and metal silos have demonstrated a good ability to maintain healthy grains even under the condition of poor drying, as they limit the supply of oxygen to potential storage pests and fungi. This will subsequently reduce the risk of aflatoxin contamination by *A. flavus* carried in grains from the field into storage facilities. Additionally, regular drying of grains to keep the moisture below 13% could prevent the proliferation of highly aflatoxigenic fungi carried from the field into storage and subsequent accumulation of high amount of aflatoxins during home storage. 

For the development of effective pre-harvest biological control, accurate characterization of non-aflatoxigenic isolates is the first step [37]. The higher proportion of non-aflatoxigenic isolates sampled in this study offers a large pool of biocontrol candidate isolates with superior traits for pre-harvest biological control of aflatoxins. The next step is testing the abilities of candidate biocontrol strains to inhibit aflatoxin production. This study showed that all of the non-aflatoxigenic isolates tested greatly reduced the level of AFB1 when co-inoculated with highly aflatoxigenic isolate A348. Additionally, this study proved that *A. tamarii* isolates were more effective at reducing aflatoxin levels than non-aflatoxigenic *A. flavus*. Five *A. tamarii* and three non-aflatoxigenic *A. flavus* isolates reduced the level of AFB1 by over 95% for the highly aflatoxigenic isolate A348. Comparatively, some members of *A. tamarii* showed the highest level of AFB1 reduction when co-inoculated with highly aflatoxigenic isolate A348 compared to most isolates of *A. flavus*. This is not the first study evaluating the role of *A. tamarii* in the control of mycotoxins; other studies have demonstrated the effect of *A. tamari* on the growth inhibition of *A. ochraceus* and subsequent reduction in the level of Ochratoxin A [38]. A similar observation was made in this study, which highlights the potential for *A. tamarii* as effective pre-harvest biocontrol agents and thus expands the pool of candidate strains for the purpose of aflatoxin mitigation. In, terms of incidence, *A. tamarii* isolates were not abundant compared to *A. flavus* isolates. Whether the limited distribution reflects the effectiveness of *A. tamarii* to colonize different staple crops in fields and displace aflatoxigenic *A. flavus* fungi is not well known.

Therefore, isolates of *A. tamarii* and non-aflatoxigenic *A. flavus* should be evaluated individually or in combination to obtain the best-performing candidates in terms of their potential to reduce aflatoxins in cereal fields and during grain storage in Uganda. Additionally, future studies should include the impacts of these strains on moderate- and low-level aflatoxin producers, who might experience complete inhibition of aflatoxin production (and possibly other toxic secondary metabolites). Adoption of suitable agents for pre-harvest biocontrol among famers would fulfill the recommendation put forward by Aflatoxin Prevention and Control Project (APPEAR) of the East Africa Community (EAC) that calls for member states to actively participate in controlling aflatoxin through the adoption of biological control [39]. The APPEAR policy has been adopted and implemented by Kenya through the introduction of KE01TM biopesticide products for controlling aflatoxin in the field crops. 

## 4. Conclusions

Most prevalent *Aspergillus* species (most of them were non-aflatoxigenic) isolated from stored household foods were *A. niger*, *A. flavus*, and *A. tamarii*. The proportion of *A. flavus* isolates was higher compared to the other species. Among the aflatoxin producers, many were capable of producing a large amount of AFB1, which is considered the most carcinogenic aflatoxin. Only one isolate could also produce aflatoxins G1 and G2. From the results, some of the non-aflatoxigenic *A. flavus* and *A. tamarii* caused significant reductions in the levels of AFB1 when co-inoculated with aflatoxigenic *A. flavus*. Therefore, *A. tamarii* isolates, in addition to commonly used *A. flavus* isolates, need to be evaluated for their potential as pre-harvest biocontrol agents.

## 5. Materials and Methods

### 5.1. Study Area

**Figure 5:**
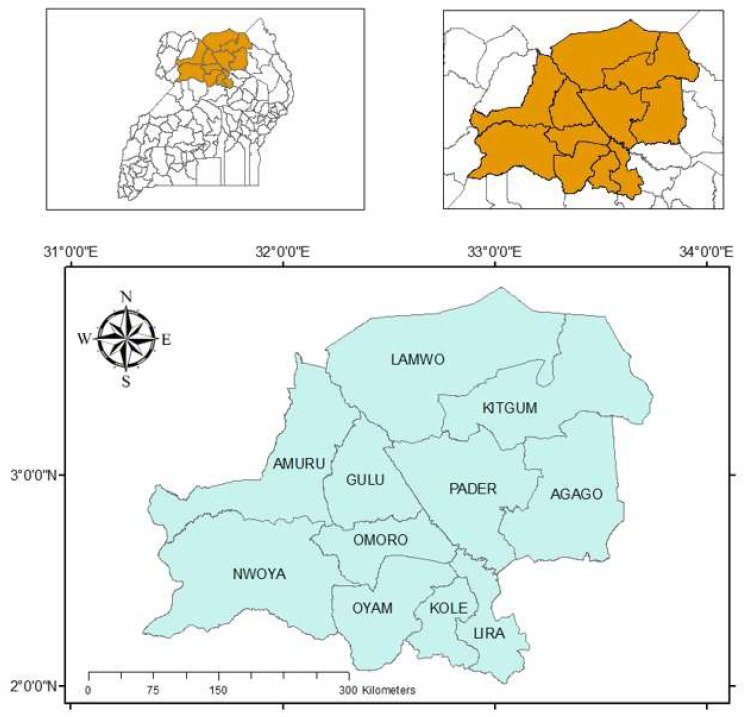
Map of Uganda (top panels) showing the 11 districts (colored brown) where sampling occurred. The names of these districts are displayed in the bottom panel.

Uganda is a landlocked country located within the eastern part of equatorial Africa. The landscape architecture is mostly a low-lying plateau with altitudes varying from 1000 to 1400 m above sea level. The temperature is particularly warm with an average of 20 °C to 25 °C depending on the months and seasons. The mean annual rainfall in most parts of Uganda varies between 500 and 2000 mm per year. In the northern part of Uganda, the period from December to March is a bit warmer with little rainfall with temperatures reaching 30 °C or more. This period is referred to as the dry season, which is due to the warm air current that comes from the dry areas of Sudan and Eritrea. The rainy season is less warm but more humid due to moist air currents that normally flow from DR Congo. Northern Uganda usually received a unimodal rainfall peak occurring between June to August compared with the central region that has a bimodal rainfall peak. In Uganda, the dry season is hottest and driest which makes it peculiar compared with other tropical countries in which the dry season is less warm. During dry seasons, very limited cultivation takes place due to limited rainfall. Meanwhile, most crops are grown during rainy seasons due to plenty of rain water. The study was conducted in northern Uganda in the following districts: Kole, Oyam, Lira, Gulu, Kitgum, Pader, Lamwo, Agago, Nwoya, Omoro, and Amuru (Figure 5). The number of samples collected from each district were Gulu (*n* = 71), Nwoya (*n* = 52), Kitgum (*n* = 67), Kole (*n* = 60), Oyam (*n* = 72), Lamwo (*n* = 63), Omoro (*n* = 83), Lira (*n* = 87), Pader (*n* = 64), and Amuru (*n* = 59).

### 5.2. Sample Types

The following cereal crops (maize, sorghum, millet, and rice), oil crops (groundnut and sesame), and root and tuber crops (cassava) are commonly grown by most people in northern Uganda and are utilized regularly. Most of these food types are frequently stored after harvest for a continuous supply of food throughout the year. The stored food from the above-mentioned crops was collected from the households for fungal isolation. The fungi were isolated from rice (*n* = 63), maize (*n* = 207), millet (*n* = 87), sorghum (*n* = 137), groundnut (*n* = 96), sunflower (*n* = 14), sesame (*n* = 71), cassava (*n* = 3).

### 5.3. Isolation and Characterization of Aspergillus Isolates from Household Grains

A total of *n* = 5 grains/seeds per sample were randomly picked and surface sterilized using ethanol: water (70:30 *v*/*v*) at least for 3 min. The ethanol residue on the surface of the grains was rinsed two times with distilled water. The surface sterile grains/seeds were seeded on Potato Dextrose Agar (PDA, 40 g/L) media and incubated at 30 °C. The fungal growth was identified based on the colony morphology which was then sub-cultured on a new PDA media [40]. The sub-cultured isolates were grown at 30 °C for ten days to generate spores. The spores were harvested using autoclaved distilled water and sieved through cheesecloth. The filtered spores were serially diluted and plated on PDA to generate single-spore colonies. A hyphal strand from each single spore was isolated then transferred to new PDA and grown until sporulation. The fungal species were tentatively identified and grouped based on colony color [40]. The spores were harvested and sieved through cheesecloth to form a stock solution for each isolate. The concentration of the spore suspensions was determined using a hemocytometer and diluted to a final concentration of 4 × 106 spores/mL.

### 5.4. Molecular Confirmation of Species Identities

Molecular confirmation of species identities was performed using primers CF1 (5′-AGGCCGAYTCTYTGACYGA-3′) and CF4 (5′-TTTYTGCATCATRAGYTGGAC-3′) that targeted the calmodulin gene [41]. The PCR mix consisted of the following reagents: 5 µL of GoTag PCR-buffer (5 X), 1.25 µL of dNTP (10 mM) solution mix, 1 µL (5 nM) forward primers, 1 µL (5 nM) reverse primers, 0.125 µL of GoTaq polymerase (5 U/µL), 2 µL of DNA templates, and 14.625 µL of water. PCR amplifications were performed using SimpliAmp™ Thermal Cycler (Applied Biosystems™ by *life* technologies™) with the following amplification conditions: initial denaturation at 95 °C for 3 min (1 cycle), 35 cycles of 1-min actual denaturation at 94 °C, 30 s of primer annealing at 50 °C, and 45 s of extension at 72 °C. Final extension at 72 °C for 5 min. The quality of the amplifications was determined by gel electrophoresis using 1% agarose gel. The PCR products were purified using MicroElute^®^ Cycle-Pure Kit (www.omegabiotek.com, accessed on 19 October 2021) and the concentration of final purified products was adjusted to a range of 10–30 ng/µL. Unidirectional sequencing of the amplicons with forward primer was done using Sanger technology. The raw sequence reads were cleanup by trimming to eliminate sequencing primers and regions of low signal intensity that occur at the beginning and at the end of the sequence reads. Nucleotide sequences were BLAST queried against the fungal collection within the NCBI database. The organism in the NCBI database with the highest percent identity to query isolates, and which was most represented among the top 100 BLAST hits generated, was used to confirm the taxonomy of the isolates.

### 5.5. Phylogenetic Tree Construction

Multiple alignments of nucleotide sequences and phylogenetic tree construction were done in MEGX [42]. ClustalW algorithm with Gap Opening Penalty (GOP) set at 15.00 and Gap Extension Penalty (GEP) set at 6.66 was used to make multiple alignments. Kimura 2-parameter with discrete gamma distribution (+5) was the best DNA model that described the nucleotide substitution pattern of our sequences. The phylogenetic tree was constructed using maximum likelihood method with a bootstrap iteration value of 1050.

### 5.6. Chemical Profiling of Isolates for Aflatoxin Production

The ability of the *Aspergillus* isolates to produce aflatoxins was determined by growing them on Yeast Extract Sucrose (YES), Potato Dextrose Agar, and on 50 g of maize grains for 10 days at 30 °C [43]. Aflatoxins were extracted from each substrate with 20 mL of methanol: acetonitrile (60:40 *v*/*v*) in a clean glass bottle. Aflatoxin assessments were conducted using Agilent infinity 1260 HPLC (www.agilent.com, accessed on 13 December 2021). The isolates were categorized according to the presence (aflatoxigenic) or absence (non-aflatoxigenic) of peaks associated with aflatoxins, and if aflatoxigenic they were further categorized as B-aflatoxin producers or B + G-aflatoxin producers. From the results, the most aflatoxigenic (A348) isolate was selected to evaluate the performance of non-aflatoxigenic *Aspergillus* in reducing level of aflatoxin produced by aflatoxigenic *A. flavus*.

### 5.7. Testing the Biocontrol Potential of Non-Aflatoxigenic Aspergilli on Maize Grains

The ability of the non-aflatoxigenic isolates to reduce aflatoxin B1 production among aflatoxigenic isolates of *Aspergillus flavus* was evaluated on harvested maize grains (Longe 5 variety). Briefly, dry grains were soaked in distilled water for 2 h and then autoclaved at 121 °C to kill any pre-existing spores or fungal mycelium within the maize kernels. For each treatment, a total of 50 g of the autoclaved maize grains were measured in a sterile 400 mL container with a lid. A 50 μL solution of normalized spores (4 × 10^6^ spores/mL) representing both aflatoxigenic and non-aflatoxigenic isolates (ratio 1:1 *v*/*v*) were inoculated onto the maize grains. For positive controls, 50 g of the grains were inoculated with 50 μL of aflatoxigenic A348 spores and water in a ratio of 1:1 *v*/*v*. For negative controls, the maize grains were inoculated with 50 µL of autoclaved distilled water. After inoculation, the grains were gently shaken to allow uniform mixing of spores on the surface of maize grains. The experimental units were the different non-aflatoxigenic isolates of *Aspergillus* section *Flavi*. Each experimental unit was replicated four times. The experiments were incubated at 30 °C for 10 days. The percent reduction of aflatoxin B1 by non-aflatoxigenic isolates was calculated as given in Equation (1).

(1) $$\mathrm{Percent}\;\mathrm{reduction}\;\mathrm{of}\;\mathrm{AFB}1=\frac{\mathrm{mean}\left(\mathrm{AFB}1_{\mathrm{aflatoxigenic}}\right)-\mathrm{mean}\left(\mathrm{AFB}1_{\mathrm{aflatoxigenic}\;\mathrm{and}\;\mathrm{non}-\mathrm{aflatoxigenic}}\right)\times 100\%}{\mathrm{mean}\left(\mathrm{AFB}1_{\mathrm{aflatoxigenic}}\right)}$$

where:

$\mathrm{AFB}1_{\mathrm{aflatoxigenic}}\;$= mean of AFB1 in the experimental unit inoculated with only aflatoxigenic isolates.

$\mathrm{AFB}1_{\mathrm{aflatoxigenic}\;\mathrm{and}\;\mathrm{non}-\mathrm{aflatoxigenic}}\;$= mean of AFB1 in experiments co-inoculated with mixture of both aflatoxigenic and non-aflatoxigenic isolates.

### 5.8. Extraction of Aflatoxins from the Experimental Setup

After the 10th day, the fungal growth was halted by drying in the oven at 50 °C for 24 h. The maize grains were milled using laboratory mortar. Aflatoxins were extracted from each treatment using a solution of methanol: acetonitrile (60:40 *v*/*v*). Briefly, 35 mL of methanol: acetonitrile (60:40 *v*/*v*) was added to 20 g of the powder and shaken at 300 rpm for 20 min. The mixture was then allowed to settle for 10 min and filtered through Whatman number 1 filter paper. A total of 1.5 mL of the extracts was centrifuged at 12,000 rpm for 5 min. Aliquot of the aflatoxin extracts were diluted with 70% methanol.

### 5.9. Detection and Quantification of Aflatoxins

Aflatoxins were detected and quantified with an Agilent 1260 Infinity II HPLC (Agilent Technologies, Palo Alto, CA, USA) that was fitted with an auto-sampler, quaternary pump, and a fluorescence light detector (FLD). Detection was performed at an excitation wavelength of 362 nm and an emission wavelength of 455 nm. Agilent eclipse plus-C18 column (4.6 × 150 mm; 5µm particle size; Agilent Technologies, Palo Alto, CA, USA) was used as the stationary phase for separation of the aflatoxin chemotypes. Mobile phase A was comprised of 238 mg of potassium bromide and 700 µL of 4 M nitric acid and adjusted to 1 L with bi-distilled water. Mobile phase B was a solution of methanol [44]. Post-column derivatization of aflatoxin was achieved using KOBRA® CELL (R-Biopharm Rhone Ltd., Block 10 Todd Campus, West of Scotland Science Park, Acre Road, Glasgow G20 0XA). The sample injection volume was 40 µL, total runtime of 12 min, and a flow rate of 0.9 mL/min was used. Aflatoxin standard was supplied as a premix solution containing AFB1, AFB2, AFG1, and AFG2 at an initial concentration of 1.02 ng/µL by Romer Labs® Division Holding GmbH (Erber Campus 1, 3131 Getzersdorf, Austria). Aflatoxin standards were prepared at a concentration of 0.51 µg/L, 0.127 µg/L, 0.06375 µg/L, 0.03175 µg/L, 0.0159 µg/L, and 0.0079 µg/L. Relative standard deviation (R.S.D.) of retention time derived from injection of aflatoxins standards was used to validate the instrument [44]. The limit of detection (LOD) for the four aflatoxins were determined. The limit of detection for AFB2 and AFG2 was 0.005 µg/L and that of AFB1 and AFG1 was 0.007 µg/L.

### 5.10. Data Analysis

The proportion of *Aspergillus* species was calculated as the sum of each species divided by the sum of all the *Aspergillus* species recovered multiplied by 100. Similarly, the proportion of aflatoxigenic and non-aflatoxigenic *A. flavus* isolated was calculated as the sum of aflatoxigenic or non-aflatoxigenic isolates divided by the sum of *A. flavus* isolates multiplied by 100. Shapiro–Wilk test was used to determine the normality of aflatoxin concentration produced by different aflatoxigenic isolates and from competition experiments. Since the normality assumption for parametric tests was not fulfilled, a non-parametric Kruskal–Wallis test was used to gain insight into the effect of each non-aflatoxigenic isolate on aflatoxin production when co-inoculated with isolate A348. A post hoc Dunn test was used to find which isolates significantly differed from each other. The results are presented in form of box plots. R-Studio v4.0 was used to perform all the statistical analysis and graphing [45].

## Figures and Tables

**Table 1:** Distribution of *Aspergillus* species isolated from household food (number of isolates = 81).

	Percentage (%)		
	*Aspergillus flavus*	*Aspergillus niger*	*Aspergillus tamarii*
Cassava	0.00	1.23	0.00
Groundnuts	7.41	3.70	1.23
Maize	11.11	3.70	4.94
Millet	3.70	2.47	0.00
Rice	8.64	1.23	2.47
Sesame	13.58	4.94	1.23
Sorghum	18.52	4.94	3.70
Sunflower	0.00	1.23	0.00

**Table 2:** Aflatoxin classes produced by the aflatoxigenic *A. flavus* isolates.

Isolates	Locations	Food Types	Aflatoxin Chemotypes			
			AFG2	AFG1	AFB2	AFB1
A635	Lamwo	Sorghum	N	N	P	P
A348	Kitgum	Maize	N	N	P	P
A584	Gulu	Sesame	N	N	P	P
A490	Kitgum	Sesame	N	N	P	P
A655	Lira	Maize	N	N	P	P
A558	Pader	Maize	N	N	P	P
ASPUN1	Nwoya	Sorghum	N	N	P	P
A508	Pader	Groundnuts	P	P	P	P
A617	Gulu	Maize	N	N	P	P
A613	Pader	Rice	N	N	P	P
ASPE1	Omoro	Sorghum	N	N	P	P
A548	Amuru	Maize	N	N	P	P
A506	Oyam	Maize	N	N	P	P
A491	Kole	Sesame	N	N	P	P
A580	Kitgum	Sorghum	N	N	P	P
A507	Kole	Groundnuts	N	N	P	P
A631	Kitgum	Millet	N	N	P	P
A334	Gulu	Maize	N	N	P	P
A633	Lira	Sorghum	N	N	P	P

P = capable of producing the aflatoxin; N = not capable of producing aflatoxin.

## Data Availability

The data presented in this study are available in the Supplementary Material here.

## Supplementary Materials

The following supporting information can be downloaded at: https://www.mdpi.com/article/10.3390/toxins14050304/s1, Figure S1: Chromatogram showing aflatoxin chemotypes of aflatoxigenic isolates; Figure S2: AFB1 level from co-inoculation of aflatoxigenic isolate A348 (control) and non-aflatoxigenic *A. flavus* and *A. tamarii* on 50 g of maize grains; Excel file S1: Level of aflatoxin B1 among aflatoxigenic *A. flavus*; Excel file S2: Level of aflatoxin B2 among aflatoxigenic *A. flavus*; Excel file S3: Variation in level of aflatoxin B1 from competition after co-inoculation of aflatoxigenic and non-aflatoxigenic isolates; Excel file S4: Percent reduction in the level of aflatoxin B1 from co-inoculation of aflatoxigenic and non-aflatoxigenic isolates.
