# Supplementary material for: Characterization of Ugandan Endemic Aspergillus Species and Identification of Non-Aflatoxigenic Isolates for Potential Biocontrol of Aflatoxins
Source: Toxins (Basel). 2022 Apr 26;14(5):304. doi: 10.3390/toxins14050304 (PMC9143334; doi:10.3390/toxins14050304)
Supplement: Supplementary file 1 [file toxins-14-00304-s001.zip › Figures S1 and S2.pdf]

# Supplementary Materials: Characterization of Ugandan Endemic *Aspergillus* Species and Identification of Non-Aflatoxigenic Isolates for Potential Biocontrol of Aflatoxins

**Godfrey Wokorach, Sofie Landschoot, Amerida Lakot, Sidney Arihona Karyeija, Kris Audenaert, Richard Echodu and Geert Haesaert**

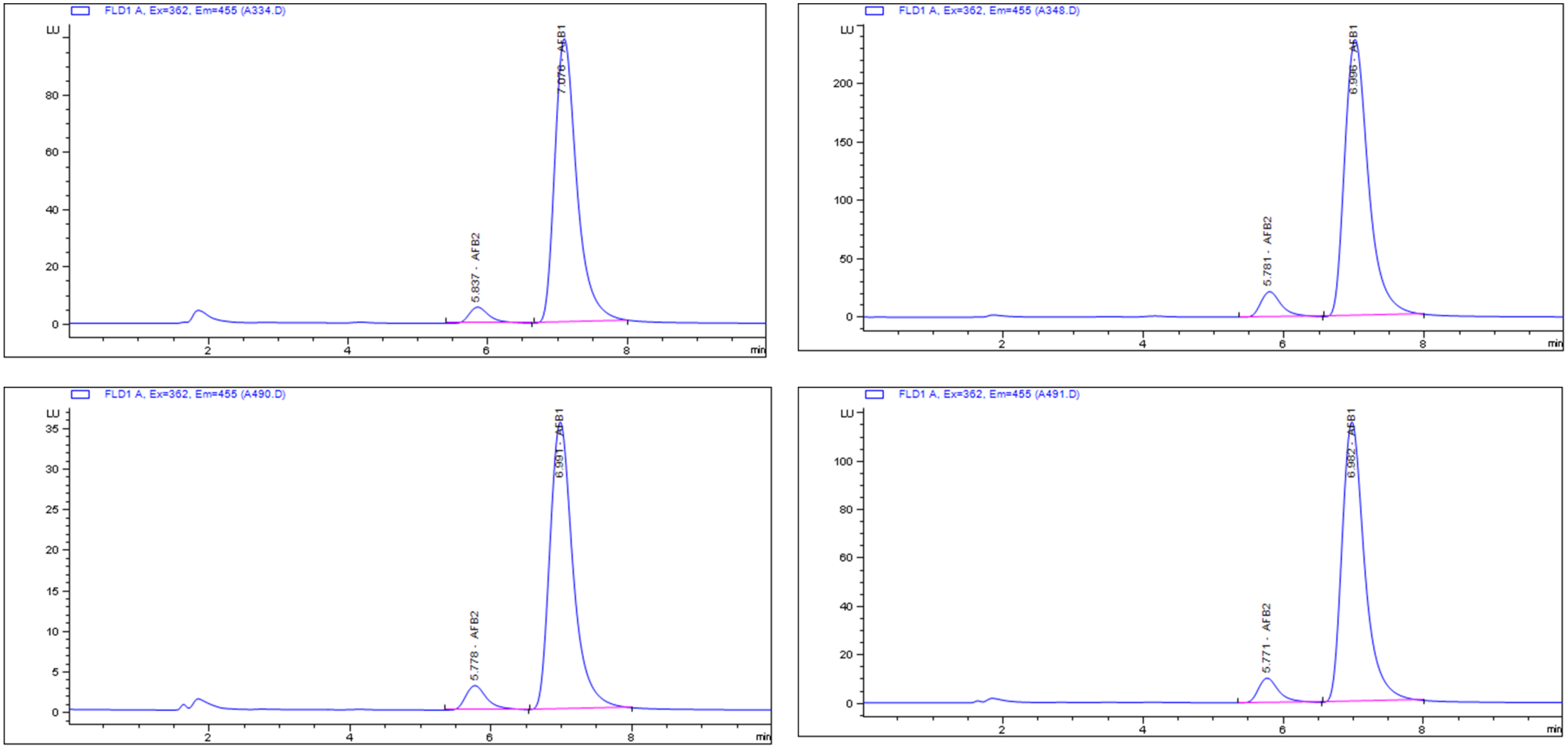


**Figure S1 A.** Chromatogram showing the peaks and retention time (RT) of aflatoxin chemotypes of aflatoxigenic isolate A344 (upper left), isolate A348 (upper right), isolate A490 (lower left) and isolate A491(lower right)

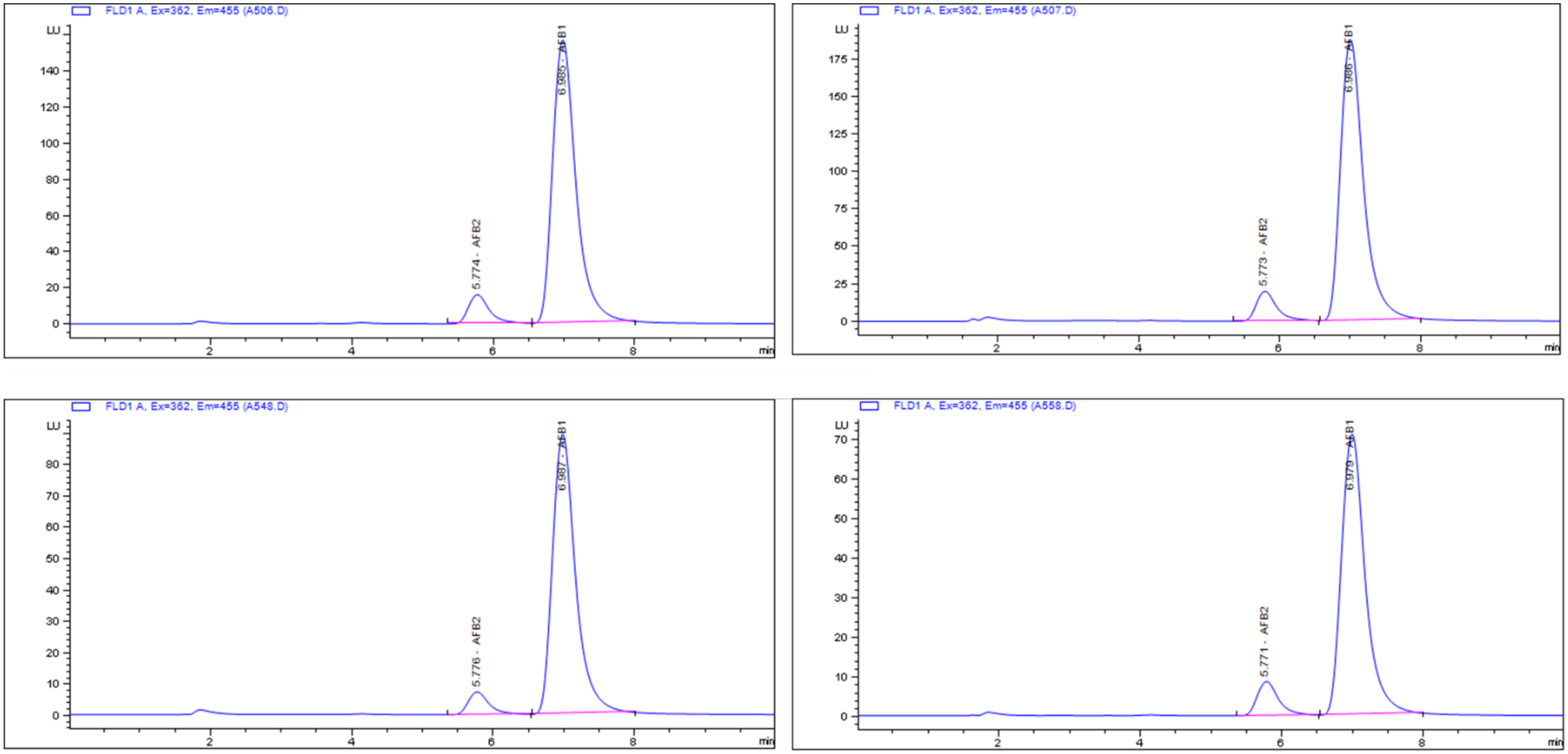


**Figure S1 B**. Chromatogram showing the peaks and retention time (RT) of aflatoxin chemotypes of aflatoxigenic isolate A506 (upper left), isolate A507 (upper right), isolate A548 (lower left) and isolate A558(lower right).

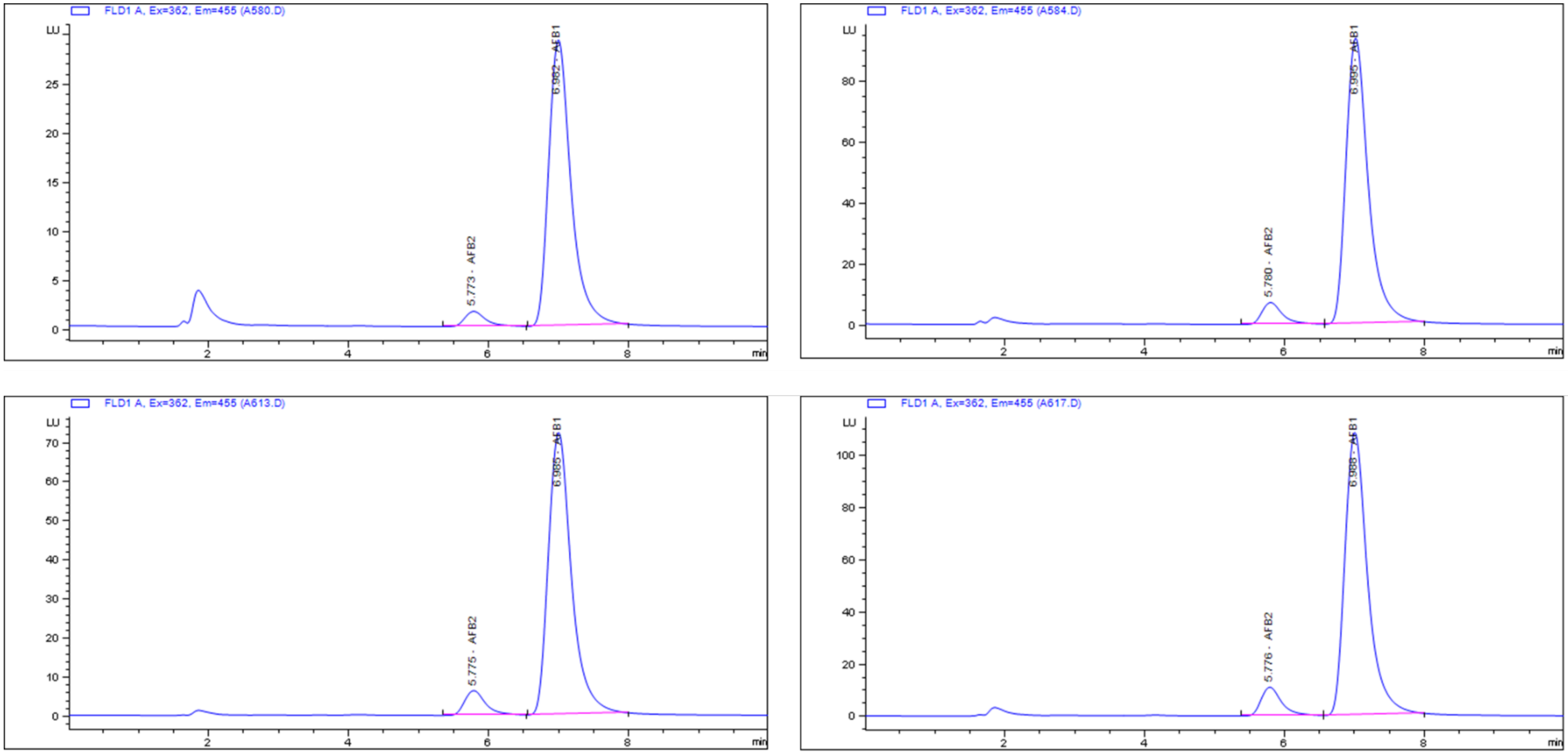


**Figure S1 C**. Chromatogram showing the peaks and retention time (RT) of aflatoxin chemotypes of aflatoxigenic isolate A580 (upper left), isolate A584 (upper right), isolate A613 (lower left) and isolate A617(lower right).

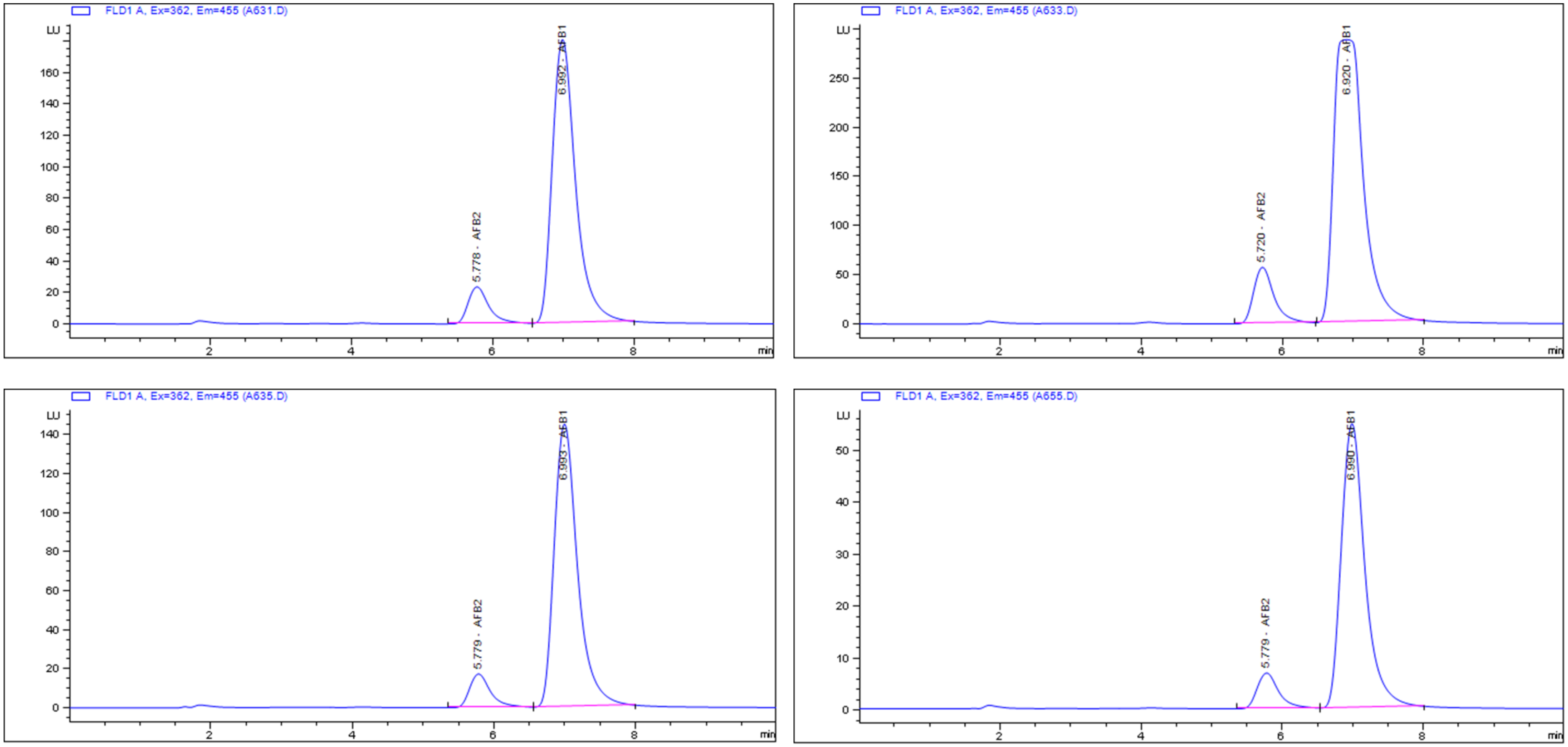


**Figure S1 D**. Chromatogram showing the peaks and retention time (RT) of aflatoxin chemotypes of aflatoxigenic isolate A631 (upper left), isolate A633(upper right), isolate A635 (lower left) and isolate A655(lower right).

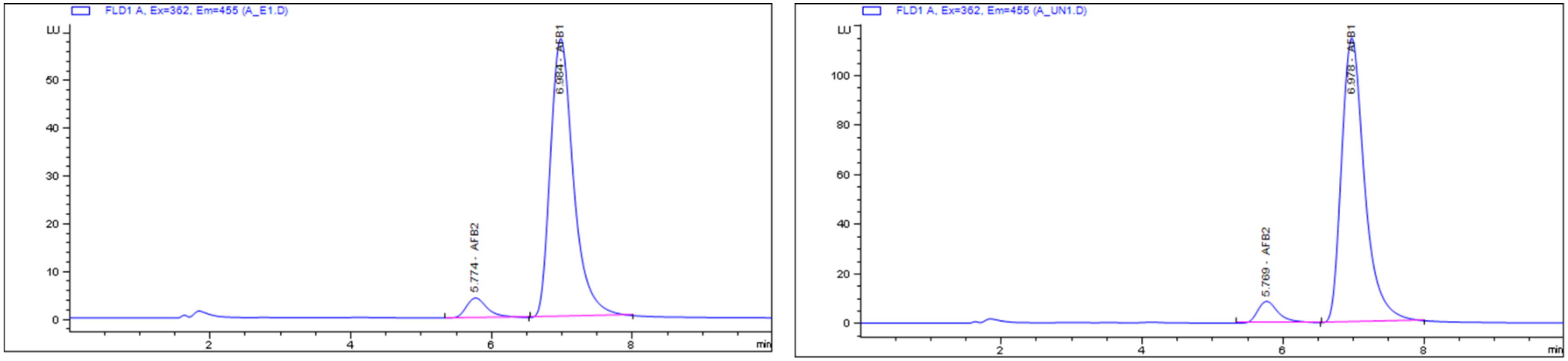


**Figure S1 E**. Chromatogram showing the peaks and retention time (RT) of aflatoxin chemotypes of aflatoxigenic isolate ASPE1 (left) and ASPUN1(right).

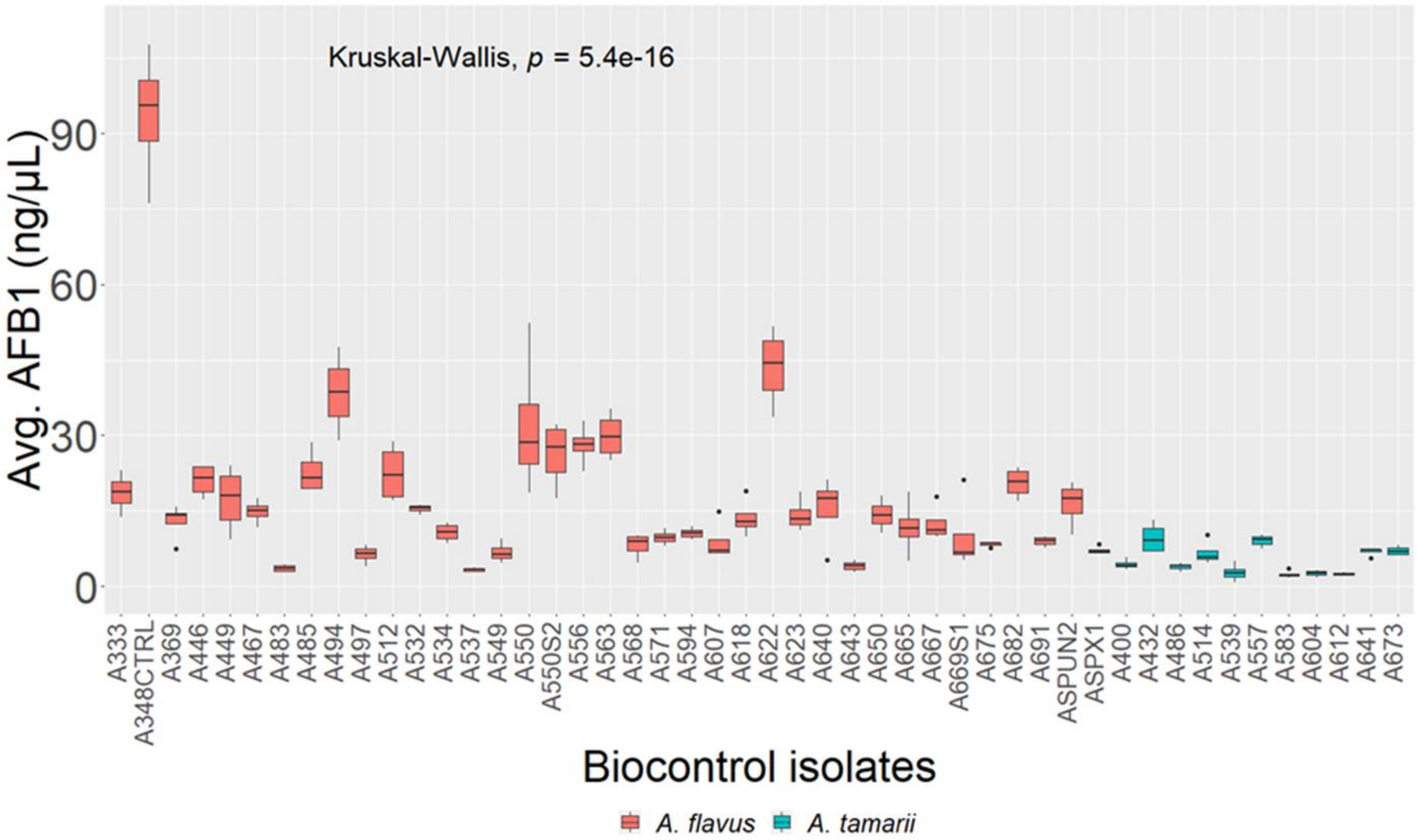


**Figure S2**. Aflatoxin B1 (AFB1) level from co-inoculation of aflatoxigenic isolate A348 (control) and non-aflatoxigenic A. flavus and A. tamarii on 50 g of maize grains.
